# CDRI-08 Attenuates REST/NRSF-Mediated Expression of NMDAR1 Gene in PBDE-209-Exposed Mice Brain

**DOI:** 10.1155/2015/403840

**Published:** 2015-08-27

**Authors:** Priya Verma, Rajaneesh K. Gupta, Behrose S. Gandhi, Poonam Singh

**Affiliations:** ^1^Department of Zoology, Mahila Mahavidyalaya, Banaras Hindu University, Varanasi 221005, India; ^2^Molecular Biology & Biochemistry Laboratory, Centre of Advance Study in Zoology, Banaras Hindu University, Varanasi 221005, India

## Abstract

CDRI-08 is a standardized bacoside enriched ethanolic extract of* Bacopa monnieri*, a nootropic plant. We reported that CDRI-08 attenuated oxidative stress and memory impairment in mice, induced by a flame retardant, PBDE-209. In order to explore the mechanism, present study was designed to examine the role of CDRI-08 on the expression of NMDAR1 (NR1) and the binding of REST/NRSF to NR1 promoter against postnatal exposure of PBDE-209. Male mice pups were orally supplemented with CDRI-08 at the doses of 40, 80, or 120 mg/kg along with PBDE-209 (20 mg/kg) during PND 3–10 and frontal cortex and hippocampus were collected at PND 11 and 60 to study the expression and regulation of NR1 by RT-PCR and electrophoretic mobility shift assay, respectively. The findings showed upregulated expression of NR1 and decreased binding of REST/NRSF to NR1 promoter after postnatal exposure of PBDE-209. Interestingly, supplementation with CDRI-08 significantly restored the expression of NR1 and binding of REST/NRSF to NR1 promoter near to the control value at the dose of 120 mg/kg. In conclusion, the results suggest that CDRI-08 possibly acts on glutamatergic system through expression and regulation of NR1 and may restore memory, impaired by PBDE-209 as reported in our previous study.

## 1. Introduction

CDRI-08 is a standardized bacoside enriched ethanolic extract of* Bacopa monnieri* (Linn.) (BM). BM has been classified as a nootropic drug in the traditional system of Ayurvedic medicine. Preliminary study indicated that a neuropharmacological effect of* Bacopa* was due to two active saponin glycosides, bacosides A and B [1, 2]. It has been put forwarded that BM treatment enhances the cognitive functions via modulating various neurotransmitters such as acetylcholine (ACh), serotonin (5-hydroxytryptamine, 5-HT), gamma amino butyric acid (GABA), glutamate, and dopamine [3–6]. Furthermore, CDRI-08 is found to attenuate the diazepam, N*ω*-nitro-L-arginine (L-NNA) and 1-(m-chlorophenyl)-biguanide (mCPBG)-induced memory impairments [7–9]. Another study suggested that CDRI-08 reduces hypobaric hypoxia-induced spatial memory impairment [10]. Recently, we have reported that CDRI-08 significantly attenuates alterations in the oxidative status in frontal cortex and hippocampus and spatial memory behaviour following postnatal exposure of 2,2′,3,3′,4,4′,5,5′,6,6′-decabromodiphenyl ether (PBDE-209) in male and female mice [11, 12]. However, molecular mechanism of action of CDRI-08 against PBDE-209-induced memory impairment is unexplored.

PBDE-209, a highly brominated congener of polybrominated diphenyl ether (PBDE), containing 10 bromine atoms, is the most widely used congener of the PBDEs. It has good thermal stability and thus requires in smaller amount to be used as flame retardant in different types of industrial and consumer products [13]. It is a persistent, lipophilic and bioaccumulates in wildlife and humans and biomagnifies up the food chain [14–16]. It has been detected not only in the environment and certain foods but also in human tissue, such as adipose tissue, serum, and the breast milk with increasing levels in rapidly developing countries such as China and India [17, 18]. Recent studies indicate that in both the environment and organisms, PBDE-209 can be debrominated to lower congeners, which have higher risks of bioaccumulation and toxicity [19, 20]. Our previous study has demonstrated that exposure to PBDE-209 causes developmental neurotoxicity by interfering in the oxidant and antioxidant homeostasis that results into impaired learning and memory performances in Morris water and radial arm maze tasks [21]. Learning and memory are majorly governed by N-methyl-*D*-aspartate receptors (NMDARs), glutamate-gated cation channels that belong to a class of ionotropic glutamate receptors (iGluRs) [22]. NMDAR is a heteromeric complex containing NMDAR1 (NR1) (GluR*ζ* in mouse) subunits in various combinations with NMDAR2A-D (NR2A-D) (GluR*ε*1–4 in mouse) and NMDAR3A-B (NR3A-B) subunits [23]. Though all the subunits play crucial role in learning and memory, NR1 subunit is obligatory for NMDAR function; therefore at least one NR1 subunit is always incorporated into the receptor complex for channel activation [24, 25]. Moreover, NR1 is found in almost all neurons of the central nervous system [26] and unlike other subunits, it is expressed consistently throughout the brain development. Genetic enhancement of NR1 expression is implicated in the formation of long-term memory [27]. However, genetic knockout of the NR1 gene blocks initiation of long-term potentiation (LTP) in both hippocampus and neocortex [28]. The expression and regulation of NR1 are challenged during several neurodegenerative pathologies, though the regulation of NR1 expression during PBDE-209-induced memory deficit is not fully understood.

Proper regulation of NR1 expression and function is required for normal physiological process within the central nervous system. Deletional and mutational analyses of the rat NR1 promoter reveal the presence of highly conserved 21–23 bp DNA sequence called Repressor Element 1 (RE1)/Neuron-Restrictive Silencer Element (NRSE) cis-regulatory site on the 5′-upstream region of the NR1 gene [29]. It modulates NR1 gene expression by binding with Repressor Element Silencing Transcription Factor (REST)/Neuron-Restrictive Silencer Factor (NRSF), a 116 kDa GLI-Krüppel class C2H2 zinc finger protein expressed in all tissues and required for proper development of vertebrates. REST/NRSF is comprised of an N-terminal repressor domain, a cluster of eight zinc fingers that functions as a DNA-binding domain, a highly basic region, a repeat region, and a C-terminal repressor domain with a single zinc finger motif [30]. The binding of REST/NRSF to RE1/NRSE represses multiple neuronal target genes in nonneuronal tissues and also in undifferentiated neural precursors of the central nervous system to control the proper timing of neuronal gene expression during neurogenesis. Conversely, disruption of REST/NRSF during embryogenesis results in cellular apoptosis, aberrant differentiation, lethality, and delayed development [31]. REST/NRSF-mediated deregulation is causative factor for several pathological conditions, such as Huntington's disease [32], cancer [33], ischemia [34], seizure activity [35], and neuropathic pain [36]. Surprisingly, it is not known whether REST/NRSF-mediated dysregulation of NR1 expression has a causative mechanism in PBDE-209-induced memory impairment and CDRI-08 has capability to revert the effects of PBDE-209 via acting on the same target. Therefore, in the light of these observations, we were interested to evaluate the role of CDRI-08 on the expression of NR1 and its transcriptional regulation mediated by REST/NRSF against PBDE-209 in the frontal cortex and hippocampus of male mice.

## 2. Materials 

### 2.1. Animals

Male and female adult Swiss albino mice, weighing 25–30 g, were maintained in animal house as per the recommendations from Central Animal Ethical Committee (number Dean/11-12/CAEC/257) of the Banaras Hindu University, Varanasi, India, for care and use of the laboratory animals. They were maintained in dedicated mice colony at 12-hour light and dark schedule at 24 ± 2°C with standard mice feed (pellets) and drinking water supply* ad libitum*. Two females were housed with one male in a cage for breeding. Females were examined every morning to observe the formation of a vaginal plug. The vaginal plug-positive females were caged individually. The day of litter born from each female was designated postnatal day (PND) 0. The size of the litter was adjusted as much as possible in order to obtain litters of the same size (6–8 pups). The newborn pups were kept with the mother till the age of PND 40.

### 2.2. Chemicals

PBDE-209 (98%, CAS number 1163-19-5) was purchased from Aldrich-Chemie while the corn oil from Sigma (St. Louis, MO, USA). The standardized ethanolic extract of BM (CDRI-08), containing 58.18% bacosides, developed by the Central Drug Research Institute, Lucknow, India, was generously gifted by Professor Singh, ex-Deputy Director [37]. The primers and oligos were obtained from IDT, USA, and Eurofins Genomics India Pvt. Ltd, India. Radiolabelled *α*P^32^-dCTP was purchased from the Board of Radiation and Isotope Technology, Hyderabad, India. Analytical grade chemicals were used for all the experiments. Molecular biology grade chemicals were used wherever necessary. Chemicals and enzymes were stored at specific temperatures, diluted, and used as per manufacturers' instructions. The stock solution of PBDE-209 was prepared by mixing the compound with corn oil and sonicated for 30 minutes at room temperature. The stock solution of BM was prepared by uniformly suspending it in 5% tween 80.

### 2.3. Experimental Design

At PND 0, male pups within the same litter were randomly assigned to five treatment groups of fourteen each and treated as follows:

 Group I: control (vehicle),
 Group II: PBDE-209 (20 mg/kg),
 Group III: PBDE-209 (20 mg/kg) + CDRI-08 (40 mg/kg),
 Group IV: PBDE-209 (20 mg/kg) + CDRI-08 (80 mg/kg),
 Group V: PBDE-209 (20 mg/kg) + CDRI-08 (120 mg/kg).

The doses of PBDE-209 and CDRI-08 were selected according to Rice et al. [38] and Saraf et al. [39]. All the treatments were given orally via a micropipette with 100 *μ*L microtip at a volume of 5.0 *μ*L/gm body weight (bw) of pups from PND 3–10. The pups were sacrificed at PND 11 (neonate) by decapitation and at PND 60 (young) by cervical dislocation. Meninges and white matter were removed carefully as much as possible. Left side of each tissue was processed for RT-PCR while that of right side for electrophoretic mobility shift assay (EMSA). Frontal cortex and hippocampus were collected and stored at −80°C as these two brain regions are involved in spatial memory function [40].

### 2.4. Semiquantitative RT-PCR Analysis of NR1 

#### 2.4.1. RNA Extraction

Total RNA from the frontal cortex and hippocampus of mice at neonate and young age was extracted using TRI reagent (Sigma-Aldrich) according to its user guidance and dissolved in diethylpyrocarbonate- (DEPC-) treated water. RNA was stored at −80°C until use [41, 42]. To make sure the RNA preparation free from DNA contaminants for further experiments, the total RNA preparation was subjected to DNA-free (Ambion) treatment using supplier's manual. The RNA content was estimated by measuring the absorbance at 260 nm taking 1 Unit A_260_ value equivalent to 40 *μ*g of RNA. The total RNA was separated on agarose gel containing formaldehyde as denaturing agent in order to check the quality of the preparation. The major RNA bands were visualized under UV light and captured the images using Alpha imager software to quantify the ratio between the 28S and 18S rRNA bands.

#### 2.4.2. Reverse Transcription

For reverse transcription of total RNA, about 2.0 *µ*g of total RNA digested with DNase I was incubated with 200 ng of random hexamers, dNTPs, and M-muLV reverse transcriptase (RevertAid H Minus, 200 units, NEB) in 1X RT buffer, supplied with the enzyme, at 42°C for 1 hour. During incubation, degradation of RNA was prevented by adding 5 units of human placental RNase inhibitor. The reaction was inactivated by heating at 70°C for 10 min and after chilling on ice, the tube was stored at −80°C or directly used for the PCR reaction.

#### 2.4.3. Polymerase Chain Reaction

Expression of NR1 and *β*-actin was assessed by amplification reaction by using the following gene-specific primers 5′-CAAGTGGGCATCTACAATGG-3′ and 5′-CCCCGTACAGATCACCTTCT-3′ for NR1; 5′-ATCGTGGGCCGCTCTAGGCACC-3′ and 5′-CTCTTTGATGTCACGCACGATTTC-3′ for *β*-actin. The PCR reactions were carried out in amplification reaction system for each gene in a 25 *μ*L reaction volume containing 2.0 *μ*L of cDNA, 2.5 *μ*L of 10X Taq DNA polymerase buffer containing 15 mM MgCl_2_, 0.6 mM dNTPs mix, 3 units of Taq DNA polymerase (Bangalore Genei), and 10 pmols of forward and reverse primers. The samples were denatured at 94°C for 5 min and amplification reactions were carried out with following amplification parameters: denaturation at 94°C for 1 min, primer annealing at 53°C (for NR1) or 57°C for *β*-actin for 1 min, elongation at 72°C for 1 min per cycle. PCR amplification was performed for 32 cycles for NR1 and *β*-actin. Various amplification parameters such as Mg^2+^ and primer concentrations, temperature for denaturation, primer annealing, elongation, and the number of cycles were determined by a pilot PCR reaction for each gene separately. Further, the number of PCR cycles was optimized such that it falls in the exponential phases of each amplification reaction (data not shown). The PCR amplified products of NR1 and *β*-actin genes were separately resolved by 1.5% agarose gel electrophoresis and the gel was visualized in UV transilluminator and photographed.

### 2.5. Electrophoretic Mobility Shift Assay (EMSA) 

#### 2.5.1. Preparation of Nuclear Extract

Nuclear extract was prepared from the frontal cortex and hippocampus of mice belonging to various experimental sets following the procedure of Dignam et al. [43] with minor modifications [44]. The nuclear proteins (extracts) were estimated by Bradford method [45]. In order to quantify the sample, 0.1 mL of the suitably diluted protein extract was mixed with 0.9 mL of the stain, mixed well, and incubated for 1 min at room temperature. The absorbance was measured at 595 nm wavelength. The standard curve was plotted by mixing varying concentrations of bovine serum albumin (BSA) and Bradford reagent and the corresponding A_595_ was measured to find out the 1A_595_ value (standard value). Using the standard value obtained from above standard curve, the protein content in the experimental samples was calculated. The nuclear proteins were analyzed on 10% SDS-polyacrylamide gel electrophoresis and stained the gel in silver staining medium.

#### 2.5.2. Annealing, Labelling, and Purification of Oligonucleotides

The complementary oligos for RE1 (−140 bp to −250 bp) 5′-GCGGAGGGTGATTCAGAGGC AGGTGC-3′and 3′-CTCCCACTAAGTCTCCGTCCACGACG-5′ were annealed in a 50 *μ*L reaction volume having equimolar concentrations of each oligo in 1X TNE buffer (10 mM Tris, 100 mM NaCl, 1 mM EDTA). Further, the oligos were denatured at 95°C in a water bath for 15 min and allowed to cool down gradually. It was incubated at room temperature overnight and stored the annealed oligos at −20°C for further use in EMSA experiments. The annealed double stranded (ds) oligos were labelled by end filling technique. The 5′ overhangs were filled in 20 *μ*L volume. The reaction mixture contained 50 ng of ds-oligos, 2.0 *μ*L of 10X reaction buffer, 2.5 *μ*L of 2 mM dNTPs mix (except dCTP), 50 *μ*Ci *α*P^32^-dCTP, and 1 unit of Klenow fragment (exo-) at 30°C for 15 min. The reaction was stopped by incubation of the reaction tubes at 70°C for 10 min. Thereafter, the labelled oligos were separated from unlabelled and free nucleotides by Sephadex G-50 spun column equilibrated with 0.19 TE buffer (pH 8.0). The first eluate after centrifugation was collected and its radioactivity content was measured by Beckman LS-100 liquid scintillation counter and stored at −70°C.

#### 2.5.3. Electrophoretic Mobility Shift Assay

To analyze the interactions of transcription factors present in the nuclear extract to their corresponding promoter sequences, the electrophoretic mobility shift assay technique was used. Approximately 5,500 cpm (corresponding to 0.1–0.2 ng) of the labelled ds oligos was used for each interaction reaction. The interaction reaction was carried out in 20 *μ*L volume. The reaction mix constituted 20 *μ*g of total nuclear protein, binding buffer, 1.0 *μ*g of* Escherichia coli* sheared DNA and 0.1 ng (or 0.2 ng) of radiolabelled oligos. After that, the tubes were incubated at 22°C for 20 min for allowing the DNA-protein binding. The reaction was terminated by adding 5.0 *μ*L of 5X loading dye (6% sucrose, 2 mM Tris-Cl, pH 8.0, 0.05% bromophenol blue, 0.05% xylene cyanol FF). The interaction reaction products (samples) from various experimental sets were electrophoresed on a prerun (1 hour at 50 V) 6% nondenaturing polyacrylamide gel (acrylamide: bisacrylamide, 19 : 1) containing 0.5X TBE (Tris-borate, EDTA) buffer at 100 V (Constant) for 1 hour. At the end of the electrophoresis, the gel was transferred onto Whatman 1 M filter paper and fixed in a medium containing 10% acetic acid and 10% methanol for 15 min at room temperature. It was then covered with saran wrap and dried for 45 min at 80°C. The gel was exposed to the intensifying screen in cassette and signals for the radiolabelled DNA-protein complexes were captured in the Phsophor Imager. Later, the signal images were scanned using Alpha Imager Software for quantitation of the interactions.

### 2.6. Statistical Analysis

All the experiments were repeated three times (7 mice/age group/set). PCR amplified DNA bands and signals of complexes were quantitated using computer-assisted densitometry (Alpha-Ease FCTM software, Alpha Innotech Corporation, CA). Results represent the mean ± SEM of data obtained from three different sets of experiments. The mean ± SEM values were analyzed by SPSS (16.0) Software. All the data were evaluated with two-way analysis of variance (ANOVA) between subject factors age and treatment followed by Tukey HSD post hoc test. A difference of *P* < 0.05 was considered statistically significant for main effects; however, difference of *P* < 0.1 was considered significant for interactions.

## 3. Results

### 3.1. Analysis of NR1 in Frontal Cortex and Hippocampus

**Figure 1:**
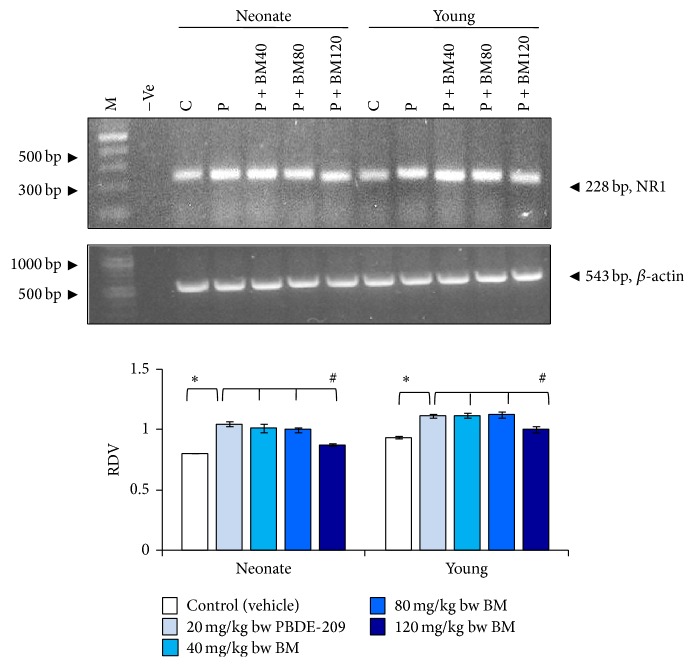
The prophylactic role of CDRI-08 (BM) (40, 80, and 120 mg/kg) against PBDE-209 (20 mg/kg) on mRNA expression of NR1 in frontal cortex of male mice at neonate and young age. M: marker (1000 bp DNA ladder), −Ve: negative control. Histograms represent cumulative data expressed as mean ± SEM obtained from three different sets of experiments. ^*^ *P* < 0.05, PBDE-209 versus control groups and ^#^ *P* < 0.05,* B. monnieri* doses versus PBDE-209 groups. RDV: relative densitometric value; C: control (vehicle); P: PBDE-209 (20 mg/kg); BM40:* B. monnieri* (40 mg/kg); BM80:* B. monnieri* (80 mg/kg); BM120:* B. monnieri* (120 mg/kg).

For expression of NR1 transcript in the frontal cortex of male mice, two-way ANOVA indicated the significant main effects of age (*F*_1,8_ 52.591, *P* = 0.000), treatment (*F*_1,8_ 240.154, *P* = 0.000), and the interaction of age × treatment (*F*_1,8_ 5.564, *P* = 0.046) by comparing the PBDE-209-exposed group with control. Further, comparison of BM-supplemented groups with PBDE-209-exposed group indicated significant main effects of age (*F*_1,16_ 43.291, *P* = 0.000) and treatment (*F*_3,16_ 16.106, *P* = 0.000); however, the interaction of age × treatment (*F*_3,16_ 0.760, *P* = 0.533) was not significant. RT-PCR data revealed that the mRNA expression of NR1 was significantly upregulated in the frontal cortex of PBDE-209-exposed neonate and young mice as compared with their respective controls (*P* < 0.05). However, following administration of 40, 80, and 120 mg/kg bw dose of BM in PBDE-209-exposed mice, a significant downregulation (*P* < 0.05) attaining the values of control in the expression of NR1 was found only at the maximum dose of BM (120 mg/kg) in the frontal cortex (Figure 1).

**Figure 2:**
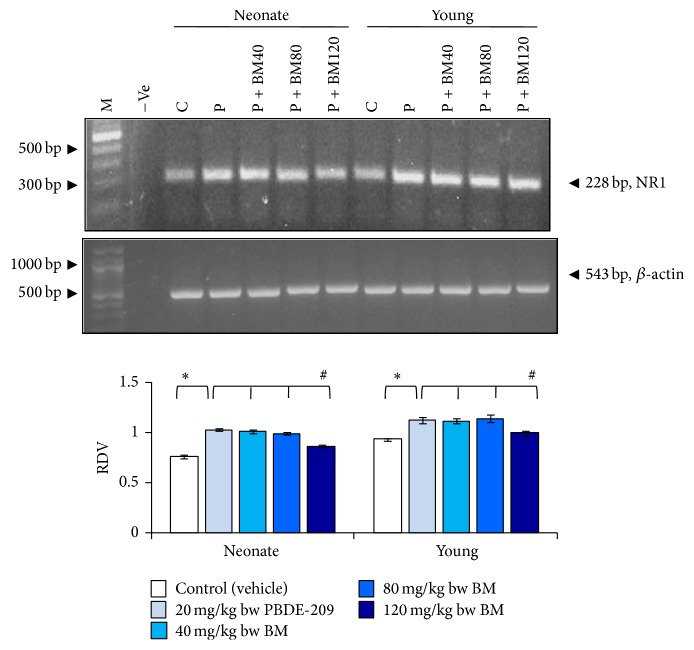
The prophylactic role of CDRI-08 (BM) (40, 80, and 120 mg/kg) against PBDE-209 (20 mg/kg) on mRNA expression of NR1 in hippocampus of male mice at neonate and young age. M: marker (1000 bp DNA ladder), −Ve: negative control. Histograms represent cumulative data expressed as mean ± SEM obtained from three different sets of experiments. ^*^ *P* < 0.05, PBDE-209 versus control groups and ^#^ *P* < 0.05,* B. monnieri* doses versus PBDE-209 groups. RDV: relative densitometric value; C: control (vehicle); P: PBDE-209 (20 mg/kg); BM40:* B. monnieri* (40 mg/kg); BM80:* B. monnieri* (80 mg/kg); BM120:* B. monnieri* (120 mg/kg).

Similarly, in the hippocampus, main effects of age (*F*_1,8_ 42.237, *P* = 0.001), treatment (*F*_1,8_ 129.276, *P* = 0.000), and the interaction of age × treatment (*F*_1,8_ 2.997, *P* = 0.122) were significant on comparison of PBDE-209-exposed group with control. Further, comparison of BM-supplemented groups with PBDE-209-exposed group indicated significant main effects of age (*F*_1,16_ 53.709, *P* = 0.000) and treatment (*F*_3,16_ 17.055, *P* = 0.000), whereas not in age × treatment (*F*_3,16_ 0.501, *P* = 0.687) interaction. Similarly, in the hippocampus of neonate and young mice, a significant restoration (*P* < 0.05) was noticed at 120 mg/kg bw dose of BMagainst PBDE-209-induced upregulation in the mRNA expression of NR1 (Figure 2).

### 3.2. EMSA of REST/NRSF with Its Cognate NR1 Gene Promoter Sequences in Frontal Cortex and Hippocampus

**Figure 3:**
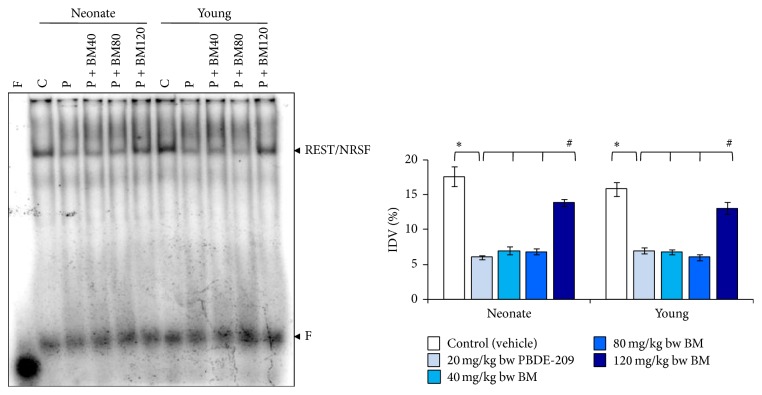
The prophylactic role of CDRI-08 (BM) (40, 80, and 120 mg/kg) against PBDE-209 (20 mg/kg) on the binding of REST/NRSF to their cognate promoter site on NR1 in frontal cortex of male mice at neonate and young age. Histograms represent cumulative data expressed as mean ± SEM obtained from three different sets of experiments. ^*^ *P* < 0.05, PBDE-209 versus control groups and ^#^ *P* < 0.05,* B. monnieri* doses versus PBDE-209 groups. IDV: %integrated density value; C: control (vehicle); P: PBDE-209 (20 mg/kg); BM40:* B. monnieri* (40 mg/kg); BM80:* B. monnieri* (80 mg/kg); BM120:* B. monnieri* (120 mg/kg).

In the frontal cortex of male mice, two-way ANOVA indicated the significant main effects of treatment (*F*_1,8_ 126.221, *P* = 0.000), whereas age (*F*_1,8_ 0.195, *P* = 0.670) and the interaction of age × treatment (*F*_1,8_ 2.281, *P* = 0.169) were not significant as compared to PBDE-209-exposed group with control on binding of REST/NRSF to NR1 promoter. Further, comparison of BM-supplemented groups with PBDE-209-exposed group on same indicated significant main effects of treatment (*F*_3,16_ 90.153, *P* = 0.000); however, the main effects of age (*F*_1,16_ 0.330, *P* = 0.573) and the interaction of age × treatment (*F*_3,16_ 1.347, *P* = 0.295) were not significant. The binding of REST/NRSF to NR1 promoter was significantly decreased in frontal cortex of PBDE-209-exposed neonate and young mice as compared with their respective controls (*P* < 0.05). Furthermore, supplementation with BM, at 120 mg/kg dose bw in PBDE-209-exposed mice, caused significant restoration (*P* < 0.05) in the binding of REST/NRSF (Figure 3).

**Figure 4:**
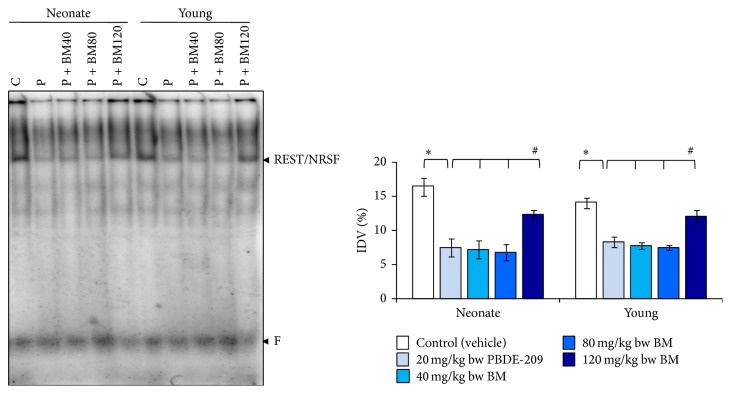
The prophylactic role of CDRI-08 (BM) (40, 80, and 120 mg/kg) against PBDE-209 (20 mg/kg) on the binding of REST/NRSF to their cognate promoter site on NR1 in hippocampus of male mice at neonate and young age. Histograms represent cumulative data expressed as mean ± SEM obtained from three different sets of experiments. ^*^ *P* < 0.05, PBDE-209 versus control groups and ^#^ *P* < 0.05,* B. monnieri* doses versus PBDE-209 groups. IDV: %integrated density value; C: control (vehicle); P: PBDE-209 (20 mg/kg); BM40:* B. monnieri* (40 mg/kg); BM80:* B. monnieri* (80 mg/kg); BM120:* B. monnieri* (120 mg/kg).

In the hippocampus, main effect of treatment (*F*_1,8_ 43.892, *P* = 0.000) was significant, while age (*F*_1,8_ 0.475, *P* = 0.510) and the interaction of age × treatment (*F*_1,8_ 2.071, *P* = 0.188) were not significant as compared to PBDE-209-exposed group with control on binding of REST to NR1 promoter. Further, comparison of BM-supplemented groups with PBDE-209-exposed group indicated significant main effect of age (*F*_1,16_ 0.545, *P* = 0.510), treatment (*F*_3,16_ 11.910, *P* = 0.000), and the interaction of age × treatment (*F*_3,16_ 0.133, *P* = 0.939). However, a significant restoration (*P* < 0.05) was noticed at 120 mg/kg bw dose of BM against PBDE-209-induced decreased binding of REST/NRSF to NR1 promoter in the hippocampus of neonate and young mice (Figure 4).

## 4. Discussion

PBDE-209, a developmental neurotoxicant, causes behavioral impairments after exposure from PND 3 to PND 10 [46] which is a critical period for brain development, called brain growth spurt period [47]. In rats and mice, the brain growth reaches its peak at PND 10; however, it continues till the first 3-4 weeks of neonatal life. This period includes axonal and dendritic outgrowth, establishment of neuronal connections, synaptogenesis, and proliferation of glia cells with accompanying myelinisation [48]. Therefore, in the present study, we have investigated the protective role of CDRI-08 against PBDE-209-intoxicated mice pups from PND 3–10, acting through REST/NRSF-mediated expression and regulation of NR1 in the frontal cortex and hippocampus. From the present findings, it is postulated that it may be one of the mechanisms of CDRI-08 to improve memory, impaired by postnatal exposure of PBDE-209 in our previous report. It is well known that the glutamatergic system mediates activity-dependent processes in both the developing and the mature brain [49]. In particular, activation of the NMDAR subtype of glutamate receptor is required for the modulation of learning and memory functions and synaptic plasticity processes, such as LTP. Although it has been suggested that spatial learning and hippocampal LTP may be associated with a differential expression of NMDAR subunits, NR1, an obligatory subunit of NMDAR complex, is required for the proper functioning of NMDAR channel [24, 25]. On the contrary, the overstimulation and pronounced activation of NMDARs by excess glutamate binding cause an immense Ca^2+^ influx and a subsequent rise in the production of reactive oxygen species (ROS) which can weaken cellular antioxidation and conduct oxidative stress [50]. In the present study the increased expression of NR1 in frontal cortex and hippocampus of neonate and young male mice following postnatal exposure of PBDE-209 (20 mg/kg) may be attributed to increased ROS levels that results into neuronal damage and impaired learning and memory by PBDE-209 [21]. Subsequently, upregulated expression of NR1 was significantly restored by supplementation with CDRI-08 at the dose of 120 mg/kg bw in the present study. The current findings are consistent with the report of Paulose et al. [51]. According to them BM plays an important role in the alteration of glutamate receptor binding and gene expression of NR1 in hippocampus of pilocarpine-induced epilepsy in rats. Bacosides present in the CDRI-08 are nonpolar glycosides; lipid-mediated transport may facilitate the bacosides to cross the blood–brain barrier by passive diffusion, which possibly act on the neurotransmitter system [52]. Considering the interaction of multiple neurotransmitters involved in learning and memory network, CDRI-08 also acts on the serotonergic system and the elevated level of 5-HT and upregulates the expression of 5-HT3A receptor, which possibly interacts with the cholinergic system [9, 53, 54]. Therefore, in the present study, it is hypothesized that CDRI-08 may act through glutamatergic system by supplementing CDRI-08 at the dose of 120 mg/kg. From the data of Zhou et al. [55], it is concluded that CDRI-08 also significantly attenuated the L-NNA-induced anterograde amnesia and partially reversing L-NNA-induced retrograde amnesia.

In concurrence with the expression of NR1, binding of REST/NRSF respond differentially to the exogenous exposures as the present study suggested decreased binding of REST/NRSF to cognate promoter sequence of NR1 in response to postnatal exposure to PBDE-209 in the frontal cortex and hippocampus of neonate and young male mice. It was significantly restored following supplementation with CDRI-08 (120 mg/kg) in PBDE-209-exposed mice indicating the steady level of NR1 expression. It has been reported that REST/NRSF dysregulation is implicated in some pathological disorders of the nervous system, such as global ischemia and epilepsy [34, 35]. To our knowledge, we provide here the first evidence that REST/NRSF is involved in PBDE-209-induced neurotoxicity. REST/NRSF functions as a transcriptional repressor of neuronal genes [56, 57]. The REST/NRSF binding motif termed RE1/NRSE is located approximately −140 to −250 bp 5′ of the initiation site of NR1 mRNA [58].

Recently, studies of neuronal gene expression have revealed a negative regulatory mechanism by which the RE1/NRSE element interacts with the active suppressor REST/NRSF. Since REST/NRSF mRNA is expressed at a high level in the embryonic brain and becomes reduced in the developing brain during the neonatal period, it is proposed that regulation of this protein plays a key role in activation of the neuronal genes during the brain development [56, 57]. REST/NRSF blocks transcription of a gene when the RE1/NRSE is located upstream or downstream of the open reading frame in either orientation. REST/NRSF is identified as a silencing element of the genes encoding SCG10 and type II sodium channel [56, 57]. Contrary to REST/NRSF pattern, the expression of NR1 mRNA remains at a low level in embryonic brain and undergoes a robust increase in the brain during the neonatal brain development [59]. This suggests that the increased binding of REST/NRSF to NR1 promoter causes reduced expression of NR1 and vice versa. REST/NRSF silences the expression of its target genes by its two independently acting repressor domains at the N- and C-termini. The N-terminal repressor domain of REST/NRSF has been shown to recruit some corepressors such as mSIN3 and histone deacetylases (HDACs) into the vicinity of the promoter. Histone deacetylation leads to a more compact chromatin that prevents accessibility of transcription factors. The C-terminal repressor domain (CTRD) of REST/NRSF has been shown to interact with at least one factor, the transcriptional corepressor CoREST that may serve as a platform protein for the recruitment of molecular machinery that imposes silencing across a chromosomal interval [60, 61].

The findings of the current study and our previous reports suggest that the REST/NRSF-mediated increase in NR1 expression observed in this study could indeed underlie the damaging effect of PBDE-209 on learning and memory. Furthermore, CDRI-08 has capability to improve memory possibly via acting through NR1 and its regulation especially by REST/NRSF-mediated regulation. This might be one of the mechanisms of CDRI-08 for the enhancement of memory.

In conclusion, our present findings provide insights of a molecular mechanism of bacosides enriched ethanolic extract of BM (CDRI-08) in improvement of memory against PBDE-209-induced impairment and suggest REST/NRSF as an attractive molecular target in CDRI-08-mediated therapy. Since REST/NRSF acts as negative regulator of NR1 gene, it maintains the steady level of expression of NR1 by binding of REST/NRSF transcription factor to cognate sequence of NR1 promoter. Thus CDRI-08 has capability to reinstate the expression and regulation of NR1, impaired by postnatal exposure of PBDE-209. Future study may need investigations on the role of CDRI-08 on downstream signaling mechanisms of memory consolidation.
